# No evidence of subgroups found in amphetamine consumers in Iran

**DOI:** 10.1007/s40211-018-0259-0

**Published:** 2018-03-07

**Authors:** Atireza Bananej, Sabine Völkl-Kernstock, Otto Lesch, Henriette Walter, Katrin Skala

**Affiliations:** 1 Department of Psychiatry and Psychotherapy, Medical University of Vienna, Vienna, Austria; 2 Department of Child and Adolescent Psychiatry, Medical University of Vienna, Waehringer Guertel 18–20, 1090 Vienna, Austria

**Keywords:** Amphetamines, Addiction, Subgroups, Amphetamine, Abhängigkeit, Untergruppen

## Abstract

Amphetamine type substances are the second most commonly consumed illicit drug type and their use is an important contributor to the global burden of disease. This investigation set out to determine whether, similar to alcohol or nicotine addiction, subgroups of consumers can also be found in amphetamine addicts. 204 consumers of methamphetamine only (*n* = 50) or both methamphetamine and heroin (*n* = 154) have been investigated in Mashhad, Iran by means of “Lesch Alcoholism Typology”. No significant differences in consumption pattern or age of onset have been found between the different types. Many subjects, however, reported symptoms of anxiety (n=78) or depression (*n* = 129) prior to drug use. These findings highlight the need for high quality epidemiological studies further addressing this issue.

## Zusammenfassung

Substanzen aus der Gruppe der Amphetamine sind die weltweit am zweithäufigsten konsumierten illegalen Substanzen, ihr Konsum trägt in hohem Ausmaß zur weltweiten Morbiditätslast bei. Im Rahmen dieser Studie haben wir untersucht, ob bei Amphetaminabhängigen, ähnlich wie bei Alkohol- oder Nikotinabhängigen, spezifische Untergruppen gefunden werden können. In Mashhad (Iran) wurden 204 Konsumenten von Methamphetamin (*n* = 50) oder Methamphetamin und Heroin (*n* = 154) mittels „Lesch Alkoholism Typology“ untersucht. Wir fanden keine signifikanten Unterschiede in Bezug auf Konsummuster oder Alter bei Konsumbeginn. Viele Patienten gaben jedoch an, vor Beginn ihres Drogenkonsums unter Angstsymptomen (*n* = 78) oder depressiven Symptomen (*n* = 129) gelitten zu haben. Diese Ergebnisse zeigen, dass es qualitativ hochwertiger epidemiologischer Studien bedarf, um sich dieses Themas weiter anzunehmen.

## Introduction

Illicit drug use is a global health concern and use of amphetamine-type substances (ATS) is an important contributor to the global burden of disease [6, 7]. The term ATS refers to various forms of stimulant drugs such as methamphetamine, amphetamine, methylenedioxymethamphetamine (MDMA, also known as “ecstasy”), methcathinone, and ephedrine. ATS are the second most commonly used type of illicit drug worldwide after cannabis (UNODC 29). In contrast with opioids, most use of amphetamine-group substances is non-injection. ATSs are most commonly snorted, smoked, or used rectally. Means of administration however varies substantially by region [11, 19]. Use of ATS can persist long term but, compared with opioids, most amphetamine-group substance use remains moderate [10]. In contrast to precursors of cocaine or opioid which can only be grown in regions with suitable climate and soil, amphetamine-group substances can be manufactured anywhere with access to the necessary ingredients. Together with the fact that precursors are widely available and usually cheap, this accounts for the increased use of ATS use worldwide. Is has to be assumed that amphetamine-group substances are manufactured in more than 60 countries [30].

While in Iran, an upper middle-income country with a population of 78 million in the west of Asia, opium and opium residues have historically been the traditional drugs of use [2], an increase of ATS use (with methamphetamine as the most frequently consumed substance) has taken place in recent years [1, 3]. While use of methamphetamines was scarce before 2005 [2, 3], investigations performed a few years later found that almost a quarter of subjects treated for opiate addiction were methamphetamine users [12] and that use had increased from 6% in 2009 to almost 20% in 2011 among former opiate addicts [14]. Despite this increase in certain subgroups, self-reported methamphetamine and ecstasy use has been found to amount to less than 1% in the general population [25].

### Subgroups of amphetamine users

Despite a growing interest in identification of biomarkers for abuse of amphetamine-group substances, only few attempts have so far been made to distinguish different groups of users. There has however been an approach to differentiate social users, who consume amphetamine-group substances to enhance interpersonal interaction [9], and functional users, whose motivation includes the desire to lose weight, improve mood, enhance work performance, and counter fatigue [4, 27]. We assume that, like alcohol addiction, amphetamine addiction also comprises heterogeneous groups consuming the drug for various effects.

The Lesch Typology of Alcoholism describes four subgroups of alcohol dependence [15]: subjects using alcohol mainly to cope with withdrawal symptoms (Type I—“biological addiction”), subjects using the drug to relieve symptoms of anxiety and tension (Type II—“anxiety-coping”), a group of consumers attempting to treat psychiatric comorbidity like depression or schizophrenia (Type III—“comorbidity”), and a group with cognitive impairment prior to the development of addiction (Type IV—“cognitive impairment”; [15–17]). The subgroups have been validated biologically and neurophysiologically (Table 1; [22, 28]). The main reason for choosing this typology has however been that clusters corresponding to those types had already been found in nicotine dependence [16].

**Table 1:** Lesch types [17]

*Lesch Type I*
No psychiatric comorbidity
Early and severe physical withdrawal of around 7 days
Long-term treatment: regular counseling
*Lesch Type II*
Anxiety, alcohol is used for conflict solving or to counteract anxiety
Mild physical withdrawal symptoms, duration around 14 days
Long-term treatment: psychotherapy
*Lesch Type III*
Underlying affective disorder, alcohol is used to relieve negative affective states
Often episodic drinking pattern
Mild physical withdrawal symptoms, duration up to 14 days
Long-term treatment: diagnosis and treatment of comorbid condition, psychotherapy
*Lesch Type IV*
Cognitive impairment (innate or subject to brain damage)
Mild physical withdrawal symptoms, duration about 7 days
Long-term treatment: self-help groups, acceptance of relapse

## Aims of the study

We assumed that, comparable to alcohol addicts, consumers of ATS also differ in the etiology of their addiction, their consumption behavior and predominantly consume for reasons like seeking stimulation, treating anxiety or depression, or coping with withdrawal. As type I includes subjects who quickly develop physical dependence to the substance in question, we did not expect to find subjects fitting this group in this sample.

In the present investigation we intended to determine whether amphetamine-dependent subjects show differences that form a pattern comparable with that of alcohol dependence. As the Lesch Alcoholism Typology (Table 2) holds several items measuring addiction per se, we applied the Lesch typology to amphetamine addicts supposing that subtyping can be a further step towards the development of individualized treatment.

**Table 2:** Excerpt of structured interview according to the Lesch Alcoholism Typology

Addictive disorder among 1st degree relatives?	(Yes/no)
Age at onset of dependence …	
*Leading substance is currently used against (multiple answers possible)*	
– anxiety – depressed mood – agitation – withdrawal symptoms – sleep disturbance – to improve well-being – other	
*Childhood development up to the age of 14:*	
Perinatal traumata:	(Yes/no)
Cerebral traumata:	(None/mild/severe)
Other cerebral diseases:	(Yes/no)
Enuresis after the age of 3:	(Yes/no)
Nail biting (>6 months):	(Yes/no)
Stuttering (>6 months):	(Yes/no)
*Other diseases*	
Depressed mood:	(None/affective mood disorder)
Sleep disturbances without alcohol intake:	(None/disturbed sleep independent of substance use or withdrawal)
Suicidal or parasuicidal tendencies:	(Never/only under influence of substance or during withdrawal/independent of substance and withdrawal)

## Methods

### Population and data collection

The study population consisted of users of methamphetamine only (*n* = 50) and users of both methamphetamine and heroin (*n* = 154). Subjects were investigated at one of the official treatment centers for addiction in the city of Mashhad, where they had at that time been under outpatient treatment for their addiction. In the group investigated, methamphetamine was the only ATS consumed by the participants. Data were collected between April and August 2014. Data collection was conducted in addition to the standard procedure performed during their routine appointment at the outpatient clinic. Every subject was investigated by the same rater.

The study was approved by the Ethics Committee of the Therapeutic Community of Mashhad (B/1395/ECTC). Informed consent was obtained from every participant.

### Measures

**Fig. 1:**
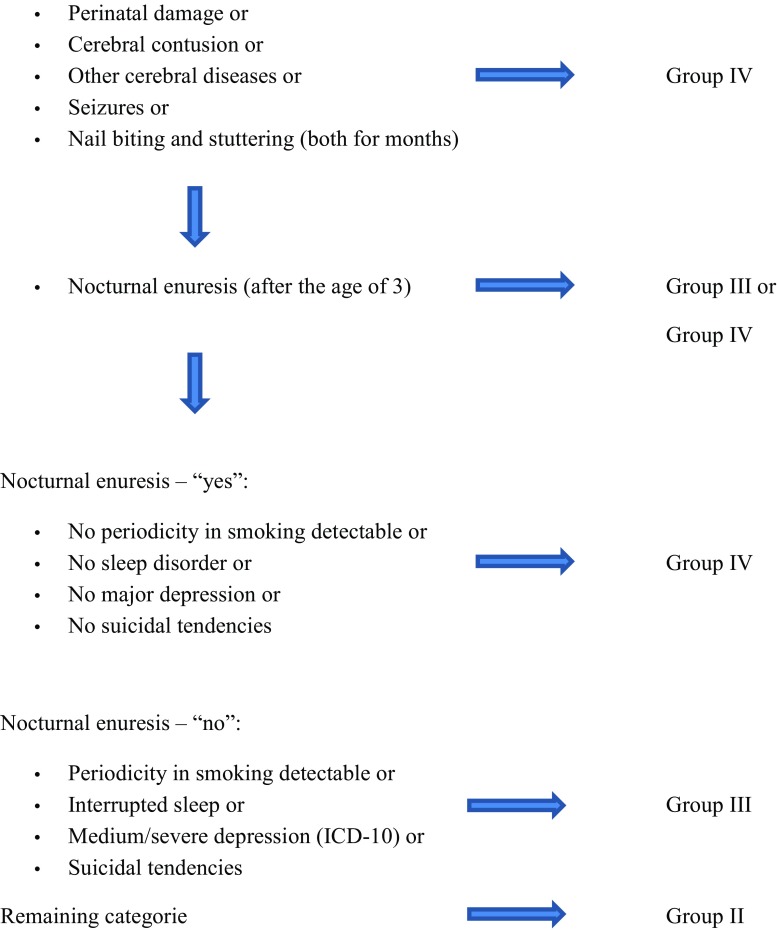
Decision tree for subgroups of amphetamine dependence

The Lesch Alcoholism Typology (LAT) software (Scholz Informatik, Vienna), version 3.2.2, was used (Table 2 and Fig. 1).

### Statistics

Chi^2^ goodness-of-fit tests were used to investigate differences between groups (type II, III, IV) concerning substance use, age of onset, and duration of consumption. As the sample was small and data were dichotomous, Spearman’s R was calculated as an additional means of interpretation.

Data analysis was conducted using IBM SPSS Statistics 21.0 software (SPSS Inc., Chicago, IL, USA).

## Results

In the present sample 39 subjects (10 using methamphetamines only and 29 consuming both heroine and methamphetamines) fulfilled the criteria of type II, 75 subjects (25 users of methamphetamine and 50 users of methamphetamines and heroine) corresponded to type III, and 90 subjects met the criteria of type IV (15 users of methamphetamine and 75 users of methamphetamines and heroine). Type I describes the quick development of physical dependence to the substance investigated. As expected, subjects corresponding type I were not present in this sample.

Of the 204 participants, 149 subjects (73%) were male and 55 subjects (27%) were female. Five subjects were younger than 20 years, 183 were between 20 and 50 years old, and 16 were 50 years and older.

### Time of treatment:

The time of treatment was less than 1 month in 82 cases and between 1 and 2 months in 47 cases, while 48 subjects underwent treatment for 2–3 months, and 25 had treatment for 3 months or more.

### Onset of consumption:

Most subjects (*n* = 179) started consumption between the ages 20 and 50, 17 subjects were less than 20, and 8 subjects were more than 50 years of age when they first consumed drugs. A total of 98 participants reported addictive disorders among immediate relatives.

### Underlying psychopathology:

While 78 participants said they had started taking drugs for their anxiety, as many as 129 participants reported to have started drug use in order to counteract states of depressed mood.

The inconsistencies of 39 participants fulfilling the criteria of Type II and 78 subjects using methamphetamine to counteract anxiety as well as 75 participants fulfilling criteria of Type III and 129 subjects trying to treat depressed mood states by means of the drug can be explained by the fact that subjects with a severe depressive disorder would occasionally suffer from anxiety (and would still be Type III) as would subjects with a diagnosis of anxiety disorder sometimes describe depressed mood states (and would still be Type II).

### Suicidal tendencies:

In all, 57 patients described suicidal tendencies independent of substance intake. Among these, suicidality was significantly more common in male subjects (*n* = 35) and in patients with neurodevelopmental problems (*n* = 90).

No significant differences in consumption pattern, time of treatment, onset of consumption, addictive disorders among relatives or suicidal tendencies have, however, been detected between the different types.

## Discussion

Amphetamine-type substances, of which methamphetamine is the most frequently used, are more widely consumed than either opioids or cocaine. They are the second most commonly used class of illicit drugs worldwide and although the prevalence rates of current methamphetamine use have been relatively stable in the US in recent years [18], consumption is increasing in east and southeast Asia and the Middle East [30].

At present, treatment options have been limited to psychosocial interventions, most of which however lack evidence [26]. Numerous classes of medication are currently under study, but none has yet been proven to be sufficiently promising [21, 23].

Assuming that subtyping can be a further step towards the development of individualized treatment, this investigation set out to determine whether there are clusters of amphetamine-dependent subjects analogous to those found in alcohol-dependent patients. Other than in nicotine dependence [16] where different clusters of consumption could be found, no such differentiation was detected in our sample of amphetamine-dependent subjects.

Interestingly, however, and comparable to alcohol addiction, many amphetamine-dependent subjects seem to use the substances in order to treat mood disorders or disturbances. While it is known that amphetamines might lead to neurological and functional impairments, facilitating the development of depressive symptomatology [8, 20, 32], we saw that one third of our sample (*n* = 78) had initially started using amphetamines to treat symptoms of anxiety, while more than half of our subjects (*n* = 129) had started using amphetamines to treat symptoms of pre-existing depression.

Although articulate affective conditions prior to onset of amphetamine dependence have, to our knowledge, not been described before, these results fit in well with results from pharmacological trials. In these mainly substances targeting dopaminergic, serotonergic, GABAergic and glutamatergic brain pathways have so far been investigated for their potential in the treatment of amphetamine addiction [31]. Among the few substances that have proven promising in reducing methamphetamine use are two antidepressants, namely mirtazapine and buproprion [24]. Like topiramate, which is also being investigated to treat amphetamine addiction, these drugs, however, show efficacy only in certain subgroups [5, 18].

So, even though articulate clusters could not be detected in this investigation it might still be useful to determine whether a patient exhibited symptoms of anxiety disorders (corresponding Type II) or depression (corresponding Type III) prior to the onset of addiction. These groups might benefit from different kinds of medications subject to their underlying condition.

The subgroups investigated here do not correspond to those already described, where consumers are classified into social users and functional users [4, 9, 27], but seem to be associated with underlying psychiatric conditions [13]. Future research that systematically addresses this gap might, thus, be able to provide support for effective treatment development for methamphetamine addiction.

## Limitations

Among the various limitations of this investigation, the cross-sectional character, the small sample size, and the combination of subjects with differing consuming patterns are the most pre-eminent. Furthermore, the questionnaire is a self-rating tool and does not allow conclusions to be drawn on definite psychiatric disorders.

## Conclusion

Although we did not identify conclusive evidence of subgroups in amphetamine addicts comparable to those described in alcohol or nicotine dependence, it was however interesting and novel to find such a large extent of reported pre-existing affective conditions prior to their drug use. These findings highlight the need for high quality epidemiological studies and closer monitoring of stimulant use in different populations.
